# Geometrically Scalable Iontronic Memristors: Employing Bipolar Polyelectrolyte Gels for Neuromorphic Systems

**DOI:** 10.1021/acsnano.4c01730

**Published:** 2024-05-28

**Authors:** Zhenyu Zhang, Barak Sabbagh, Yunfei Chen, Gilad Yossifon

**Affiliations:** † School of Mechanical Engineering, Tel Aviv University, Tel Aviv 6997801, Israel; ‡ Jiangsu Key Laboratory for Design and Manufacture of Micro-Nano Biomedical Instruments, School of Mechanical Engineering, Southeast University, Nanjing 211189, China; § Faculty of Mechanical Engineering, Technion−Israel Institute of Technology, Haifa 3200003, Israel; ∥ Department of Biomedical Engineering, Tel Aviv University, Tel Aviv 6997801, Israel

**Keywords:** memristor, polyelectrolyte, iontronics, ion transport, neuromorphic, nanofluidics

## Abstract

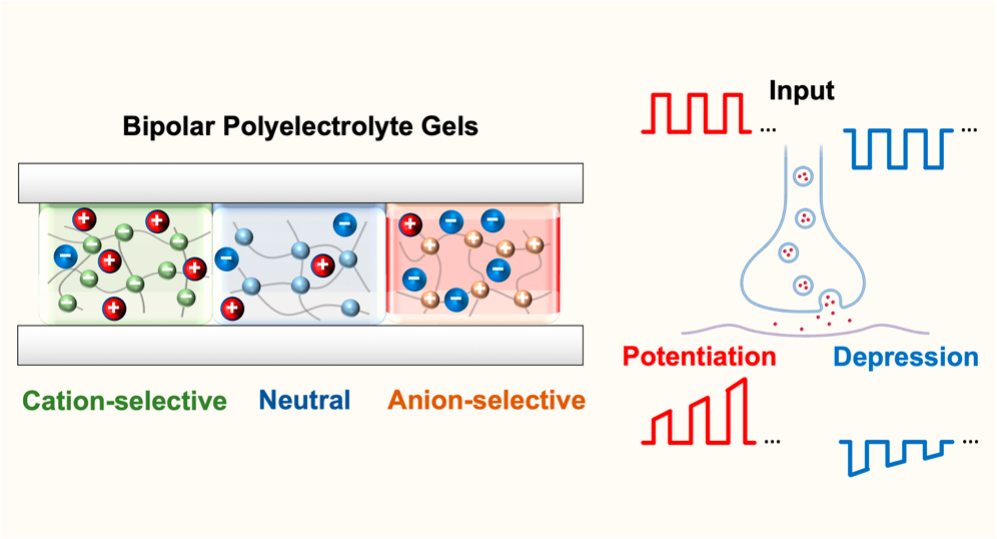

Iontronics that are capable of mimicking the functionality of biological systems within an artificial fluidic network have long been pursued for biomedical applications and ion-based intelligence systems. Here, we report on facile and robust realization of iontronic bipolar memristors featuring a three-layer polyelectrolyte gel structure. Significant memristive hysteresis of ion currents was successfully accomplished, and the memory time proved geometrically scalable from 200 to 4000 s. These characteristics were enabled by the ion concentration polarization-induced rectification ratio within the polyelectrolyte gels. The memristors exhibited memory dynamics akin to those observed in unipolar devices, while the bipolar structure notably enabled prolonged memory time and enhanced the ion conductance switching ratio with mesoscale (10–1000 μm) geometry precision. These properties endow the devices with the capability of effective neuromorphic processing with pulse-based input voltage signals. Owing to their simple fabrication process and superior memristive performance, the presented iontronic bipolar memristors are versatile and can be easily integrated into small-scale iontronic circuits, thereby facilitating advanced neuromorphic computing functionalities.

## Introduction

Neuromorphic computing, inspired by the working principles and architecture of the human brain, offers means of advancing artificial intelligence.^1,2^ When compared to the von Neumann architecture, it promises to improve energy efficiency and computational capacity, particularly for complex workloads.^3^ To this end, memristors have been demonstrated as an artificial analog to biological neuron synapses^4,5^ and have been successfully integrated in various platforms, for applications ranging from augmentation of CMOS chip functionalities^6^ to in-memory computing and sensing.^7^ Yet, the progress in this field is significantly slowed by challenges relating to performance, manufacturability, and the choice of materials.^8^

Iontronic systems capable of mimicking energy-efficient biological information processing are attracting increasing interest.^9−12^ Compared with electronic systems, iontronics can transduce and process both electrical and chemical signals via the transport of various ions and charged molecules in aqueous solutions or polymers, reminiscent of signal transmission in biological systems. Micro-/nanofluidic iontronics are usually composed of nanometer-sized fluidic structures that exhibit high ion permselectivity due to overlapping electrical double layers (EDLs).^13,14^ Recently reported advancements include nonlinear fluidic devices such as ionic diodes,^15−18^ transistors,^19,20^ and capacitors.^21^ These devices are not only pivotal to fundamental research but carry potential for various applications in energy harvesting,^22−24^ chemical sensing,^25^ electrokinetic preconcentration,^26,27^ and biohybrid interfacing.^20,24^ Specifically, iontronic nanofluidic memristors have been used to realize basic neuromorphic functions, by leveraging various mechanisms such as in-channel concentration polarization,^28−30^ ion adsorption at the nanofluidic wall,^31,32^ salt precipitation,^33^ interionic/molecular interactions,^34,35^ electrochemical reactions,^36^ and liquid–liquid interface movement.^28,29,31−38^ Such memristors are capable of producing ionic memory spanning from milliseconds^29,32,38^ to over an hour,^31,33,37^ with conductance on/off ratios varying from 2^31^ to 20.^38^

One of the key challenges of fluidic memristor development relates to achievement of optimal temporal electrical performance, characterized by a large conductance on/off ratio and long-term plasticity.^8,38^ It involves maintenance of robust depression and a potentiation state for extended periods of minutes and even hours. Achieving prolonged memory time along with large memristive current–voltage (*I*–*V*) curve hysteresis in fluidic memristors, particularly those relying on ion concentration polarization (ICP), is challenging due to diffusion, which challenges conservation of a concentration gradient over a long period of time within continuously connected fluidic systems. Robust mechanisms that have been realized for memory periods extending beyond 1 h include ion adsorption^31^ at an activated graphene nanochannel wall and salt precipitation in microfluidic polyelectrolyte gel.^33^ Because the performances of these systems are governed by the physical and chemical processes between the participating ions and the fluidic channel walls, the resulting memory times are nonscalable. They also pose challenges to manufacturing, especially with regard to integration of two-dimensional materials on miniaturized chips. Furthermore, their integration in small-scale fluidic circuits for handling complex tasks has hardly been achieved,^38^ limiting their potential applications in advanced computing systems.^16^

This work reports on iontronic memristors featuring a three-layer bipolar ion-selective hydrogel structure that enhances the control of ion transport. These memristors demonstrate scalability of memory timescales from seconds to hours, controlled by ICP within polyelectrolyte gels in a microfluidic channel architecture. The fabrication process is cost-effective, rapid, and facile, eliminating the need for photolithography and etching procedures. The experimentally realized iontronic memristors were characterized in terms of *I*–*V* hysteresis, transient current responses, and pulse signal processing capabilities. Additionally, numerical simulations were conducted to elucidate the underlying physics of the memory function in the bipolar devices. This work provides insights into the development of reliable iontronic bipolar memristors for small-scale integration into ionic circuit systems.

## Results and Discussion

### Continuous Electrical Characterizations

**Figure 1:**
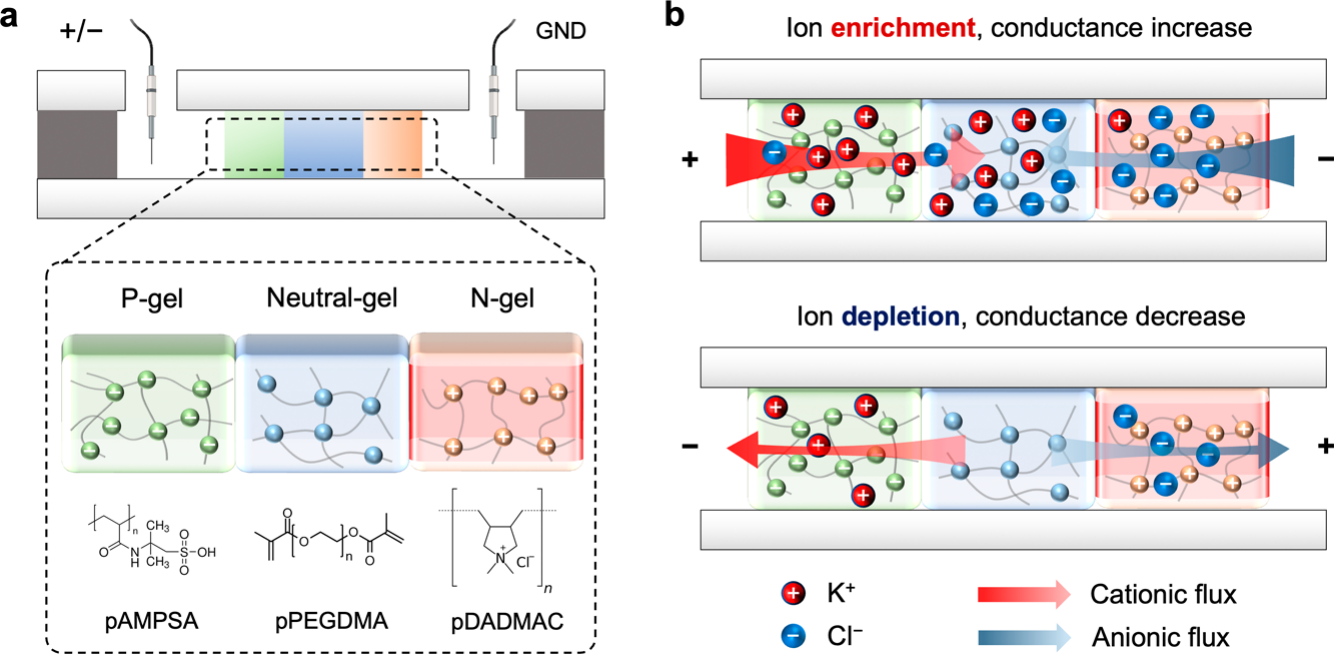
Schematics of the iontronic bipolar memristor structure and operation. (a) Fluidic memristor structure and setup. Microchannels are patterned by femtosecond laser cutting of adhesives between two glass slides. Three types of hydrogels are immobilized within the microchannels by photo-crosslinking in order to pattern and form three distinct layers of different ion-selective membranes. (b) Schematic of the working principle of ionic bipolar memristors. The red and blue spheres represent K^+^ and Cl^–^ ions, and red and blue arrows indicate the direction of cationic and anionic flux in the system, respectively.

The design of the iontronic bipolar memristors is shown in Figure 1a. A facile and robust fabrication technique was employed to rapidly prototype the memristor designs, as detailed in the Methods section and in on our previous works.^16,18^ First, a planar double-sided adhesive sheet was cut to specific patterns by a femtosecond laser (refer to geometry design in Figure S1 and the fabrication process in Figure S2). The design included a shallow middle channel to facilitate effective modulation of ion transport via in-channel ICP. The adhesive sheet with the microchannel patterns was transferred to a clean glass slide and sandwiched by another glass slide to form closed microchannels. Then, three types of polyelectrolyte gels, pPEGDMA in the middle channel (M gel) and P-type pAMPSA (P gel) and N-type pDADMAC (N gel), which are nonselective, cation-selective, and anion-selective, respectively, were patterned sequentially within the formed microchannels by UV photopolymerization using photomasks. Subsequently, the microchannels were washed and filled with the electrolytes. Two Ag/AgCl electrodes were connected to the inlet reservoirs for electrical characterization. More details are provided in Figure S2 and Methods.

Figure 1b illustrates the operational mechanism of the devices. The high-density fixed charges in the P and N gels spontaneously electrostatically attract counterions while excluding co-ions, in accordance with the induced Donnan potentials.^39^ Similar to iontronic diodes, the bipolar structure is expected to facilitate a high current rectification ratio due to enhanced control of ion transport.^12^ The M gel microchannel section features significant electrical resistance due to its narrow width and, hence, is expected to be the most sensitive region for ion conductance modulation. Initially, cations accumulate in, and anions are depleted from the P gel, with the opposite effect occurring within the N gel. When a forward voltage bias is applied, both cations and anions are driven from the interfacing microchannels into the central M gel region, accumulating over time and thus continuously increasing its ionic conductance. Eventually, the system reaches an on state as the conductance level plateaus. Conversely, when the device is reverse biased, the ionic electromigration is reversed, leading to ionic depletion within the M gel, which, in turn, exhibits high resistance, and the bipolar memristor is switched to an off state.

**Figure 2:**
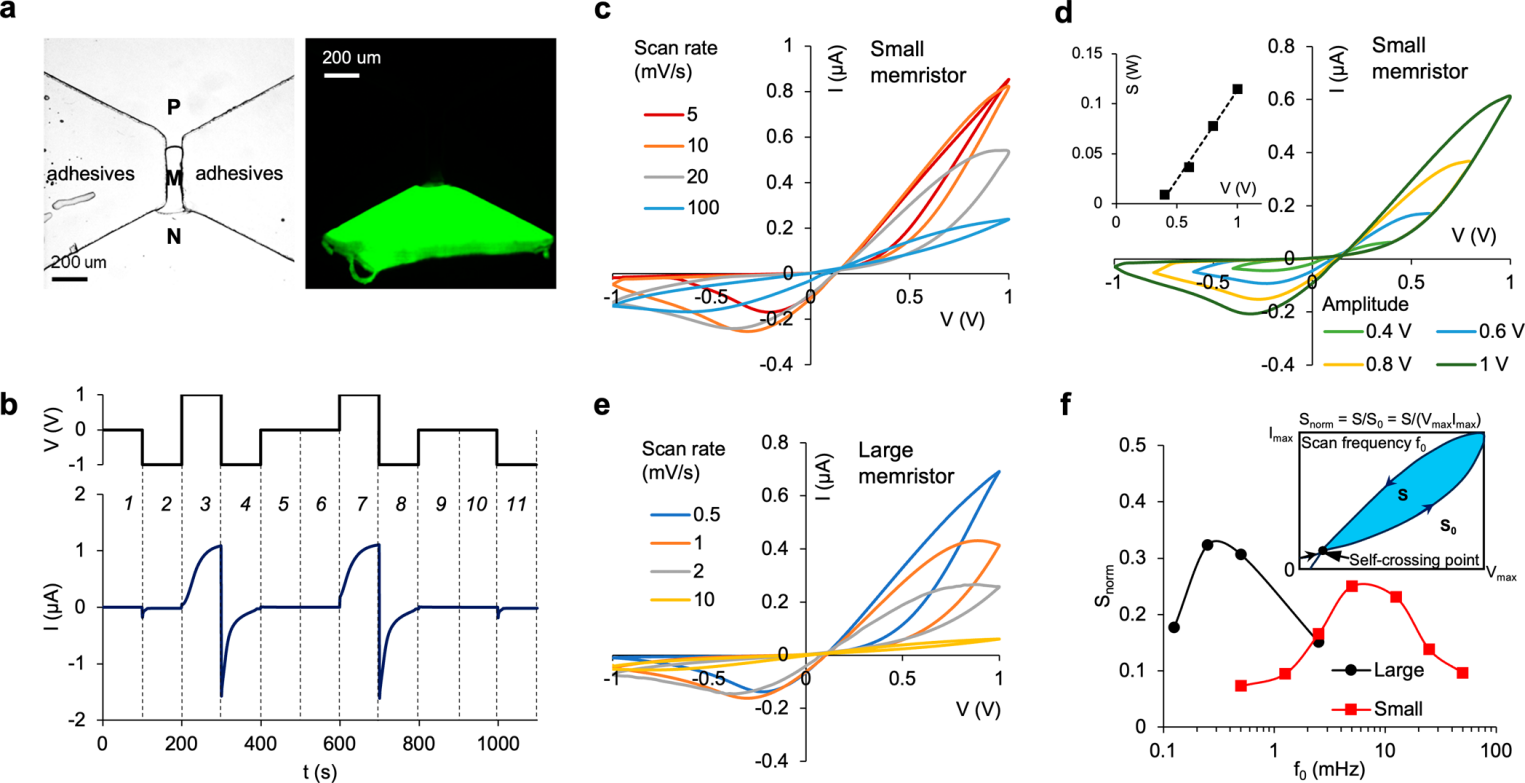
Experimental characterization by optical imaging and transient ion current measurement. (a) Bright-field and anionic fluorescence image of the “small” memristor after electrical characterizations. Left and right sides are the remaining adhesives. The middle channel was patterned with pPEGDMA gel (marked as “M”), and the upper and lower sides of the trapezoids were patterned with pAMPSA and pDADMAC gels, which are selective to cations (type “P”) and anions (type “N”), respectively. The minimum width of the middle channel was 100 μm, and its length was 500 μm. The anionic fluorescence image presents the area of the anion-selective pDADMAC gel membrane. (b) Ion current response to various voltage steps. Steps 1, 5, 6, 9, and 10 were no bias, 3 and 7 were +1 V, and steps 2, 4, 8, and 11 were −1 V, with each step lasting for 100 s. (c) *I*–*V* curves at scan rates ranging from 5 to 100 mV/s characterized on the device in (a). All curves were obtained with a constant 1 V amplitude when the current response stabilized after several AC voltage cycles. (d) *I*–*V* curves obtained at a constant scan rate of 10 mV/s but at different amplitudes. Inset: *I*–*V* hysteresis loop area at different voltage amplitudes (*V* > 0). (e) *I*–*V* curve characterized on a “large” bipolar memristor, with a middle neutral layer length of 2 mm and a width of 500 μm. (f) Normalized hysteresis loop area measured at different scan frequencies. The inset shows the definition of the hysteresis loop area at *V* > 0 and its normalization. *S* is the loop area on the right of the self-crossing point, and *S*_0_ is the area of the rectangle.

Figure 2a shows the microscope image of the “small” memristors after several days of tests. When the reservoir was kept full with working solutions (10 mM KCl), the polyelectrolyte gels maintained their original shape and functionality over a week. The boundaries between the P and N gels and the microchannel solution were practically indiscernible, yet they became clear under a fluorescence microscope after swelling in solutions with an ionic dye. Several large memristors with a middle channel length of 2 mm and a width of 500 μm and P and N gel lengths of 1 mm each were fabricated. Stepwise chronoamperometry (stepping *V* while monitoring *I* as a function of time) was employed to measure the temporal response of the ion current under a train of input steps. Figure 2b illustrates the response of an ion current to a voltage signal with 11 steps at a switching frequency of 10 mHz. Notably, when switching to a forward bias under a constant applied voltage, the initially low ionic current gradually increased with time. This is in contrast to bipolar diodes where the ionic current decreases over time when forward biased as a result of ICP at the microchannel–polyelectrolyte interfaces, wherein the overall resistance is dominated by that of the increased resistance of the depleted microchannel.^16^ Although such an ICP at the microchannel–polyelectrolyte interface should also be present in the memristors, the memristive electrical behaviors originating from the three-layer polyelectrolyte gels dominate the overall resistance. Switching voltage steps from 1 to −1 V led to rapid depletion of the accumulated ions, causing a significant reverse current peak, which was markedly more pronounced than after the transition from 0 to −1 V (Figure 2b).

The bipolar memristors demonstrated pronounced memristive hysteresis at optimal *I*–*V* scan rates. The small memristor exhibited a substantial hysteresis loop at scan rates of 10 and 20 mV/s (Figure 2c). At an elevated scan rate of 100 mV/s, the voltage application time precluded adequate ion enrichment and depletion within the M gel, which in turn resulted in a diminished conductance on/off ratio at ±1 V attributable to negligible ion concentration variation in the middle channel. On the other hand, at a reduced scan rate of 5 mV/s, ionic memory was compromised due to the inevitable ion diffusion across P and N gels. Figure 2d shows that the hysteresis behavior remained qualitatively similar with respect to the voltage amplitude at a fixed scan rate and increased with increasing voltage. Figure 2e elucidates the *I*–*V* curve obtained with the “large” bipolar memristor. It demonstrates a reduction of the optimal scan rate suitable for pronounced hysteresis by 1 order of magnitude, from 10 to 1 mV/s, achieved by changing of the microchannel geometry design. The existence of pinched hysteresis of *I*–*V* curves is one of the key characteristics of memristive system response. In the ideal memristor, the pinched self-crossing point intersects the origin because there is no memresistance change at 0 V. The slight shift of self-crossing points of the *I*–*V* curves away from 0 in Figure 2 is due to parasitic impedance effects of ion diffusion in and out of the polyelectrolyte gels. All *I*–*V* curves obtained so far for both small and large bipolar memristors can be stabilized after several scans, as shown in Figure S3c–e.

**Figure 3:**
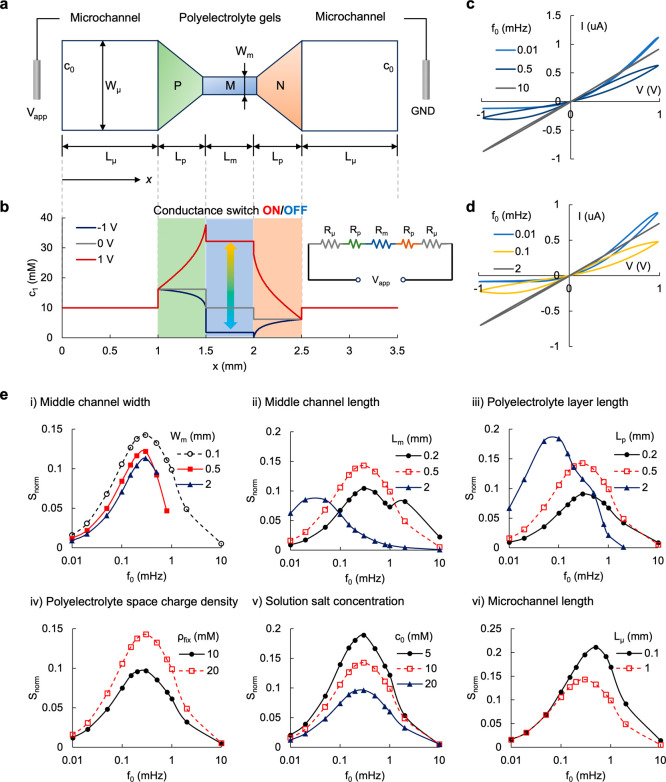
Numerical simulations of iontronic bipolar memristors using a one-dimensional model. (a) Setup of the numerical model. P, M, and N represent the P-type (i.e., cation-selective), middle electroneutral, and N-type (anion-selective) polyelectrolyte gel layers, respectively. (b) Cation concentration distributions along the *x* direction at different bias voltages at the steady state. Inset: an equivalent circuit with only resistors. (c) *I*–*V* curve response to triangle voltage scan from the model with the same geometry as in Figure 2a. A 10 mM KCl solution was employed as the boundary condition at the inlet and outlet of microchannels. (d) Same as in (c) but for the large bipolar memristor. (e) Memristor hysteretic response dependence on (i) middle channel width *w*_m_, (ii) middle channel length *L*_m_, (iii) polyelectrolyte layer length *L*_p_, (iv) polyelectrolyte space charge molar density *N*, (v) solution salt concentration *c*_0_, and (vi) microchannel length *L*_μ_. Dashed lines and empty symbols refer to the simulation results of the original numerical model of a small memristor as a reference.

Figure S3a,b shows the *I*–*V* curves for a low scan rate with a voltage range from −3 to 3 V, where maximum steady-state ion current rectifications of *I*(1 V)/*I*(−1 V) = 86.5 and 79.0 were obtained over all bias voltage amplitudes for the small and large memristors, respectively. The conductance switching ratios at the memristor operating frequencies, calculated as *I*_f_(1 V)/*I*_f_(−1 V) from the hysteresis *I*–*V* curves in Figure 2c,e, were 20.5 (small memristor, 2.5 mHz) and 8 (large memristor, 0.25 mHz), where the subscript f refers to the voltage scan frequencies, which is calculated by *f* = (voltage scan rate)/4 V. As discussed in our prior papers,^16,40^ the nonlinearity of the *I*–*V* curve at a large forward biased voltage is attributed to ICP in the microchannels. The electrical resistance of the microchannel, denoted as *R*_μ_ = *L*_μ_*W*_μ_^–1^*H*_μ_^–1^σ^–1^ (see Figure 3a), generally plays an important role in the operation of bipolar diodes under forward bias. In the small bipolar memristors (Figure 2a), the resistance ratio of the microchannel to total resistance *R*_μ_/*R*_total_ was 0.6 when forward biased and dropped below 0.01 under reverse bias, where *R*_total_ is the total electrical resistance taken from experimental measurements. Moreover, memristive behavior as an iontronic counterpart of electronic memristors^5^ only occurred with the inclusion of the intermediate M membrane layer. In comparison, a P–N bipolar diode without such an intermediate layer exhibited *I*–*V* hysteresis without the self-crossing point (Figure S3f), which is attributed to residual ICP in the microchannels.

When extending the investigation to the dynamic electrical response of the memristor, it is instructive to consider how the hysteresis loop varies with the AC voltage (of triangular waveform) frequency. Figure 2f shows the normalized hysteresis area *S*_norm_ versus voltage scan frequency *f*_0_. *S*_norm_ is the hysteresis loop area between forward and reverse *I*–*V* curves at *V* > 0 to the right of the self-crossing point normalized by *V*_max_ × *I*_max_, which refers to the maximum scan voltage and maximum measured current in a loop, respectively (Figure 2f inset). The parameter *S*_norm_ provides a quantitative assessment of the hysteresis quality in the bipolar memristors. The hysteresis loop area peaked when *f*_0_ matched the inverse of the intrinsic memory time *f*_max_ = 1/τ_m_. The experimentally measured frequencies of the maximum *S*_norm_ were *f*_max_ = 0.25 mHz for the large memristor and 5 mHz for the small memristor, corresponding to τ_m_ of 4000 and 200 s, respectively. When considering the diffusion in 2D microchannels, by Fick’s law, we can write τ = *L*^2^/4*D*,^29^ where the polyelectrolyte gel total region lengths (*L*) are 4.3 (large) and 1.5 mm (small) and the ion diffusion coefficient (*D*) is 2 × 10^–9^ m^2^/s. The estimated results are 2311 and 281 s for the large and small memristors, respectively, when assuming that the diffusion coefficient in hydrogels is the same as in bulk solution. The scalability of ionic memory with geometry, ranging from minutes to over 1 h, is reminiscent of the short-term and long-term plasticity (the ability of synapses to strengthen or weaken over time) in biological neuron systems in response to external stimuli. The performance of the device remains stable for weeks when immersed in working solutions. Leveraging the facile and robust fabrication protocol, this scalable ionic memory feature may prove essential to small-scale integration of iontronic memristor-containing devices with a broad range of memory times.

### Underlying Physics and Parametric Study by Numerical Simulations

To investigate the fundamental ion transport mechanism underlying the memristive effect, with focus on the effect of geometry and space charge of the polyelectrolyte gel, a simplified one-dimensional numerical model based on Poisson–Nernst–Planck equations was constructed (Figure 3). The microchannel width was considered by employing an effective diffusion coefficient as *D*_*e*_ = *DW*(*x*)/*W*_μ_.^41^ The fixed charged functional groups of polyelectrolyte gels were represented by fixed uniform volumetric space charge densities (for more details, see the Supporting Information (Table S1)). A concentration rectification ratio of approximately 18 (steady-state) was obtained in the small memristors at a ±1 V bias voltage, leading to effective ionic conductance on/off switching. Time-dependent simulations qualitatively reproduced the memristive hysteresis in experimental setups for both the small (Figure 3c) and large (Figure 3d) memristors.

An equivalent electrical circuit of the bipolar memristor is shown in the Figure 3b inset. The M gel section is present in the studied memristors while absent in bipolar diodes and exhibits a ratio of electrical resistance *R*_m_/*R*_total_, calculated from the electrical potential drop in Figure 3b, ranging between 0.48 (forward bias) and 0.87 (reverse bias) (Figure S5g). The change in the electrical resistance of the M gel Δ*R*_m_ = *R*_m,high_ – *R*_m,low_ was intuitively used as an indicator of steady-state ICP in polyelectrolyte gels. Hence, Δ*R*_m_/*R*_total_ encapsulates the system resistance switching between on and off states and the dynamic resistance switching correlating to the hysteresis of the ICP in M gel. Results suggested that *S*_norm_ increases with decreasing *W*_m_ (width of the central neutral membrane) (Figure 3e, scenario i), while the scan frequencies at which *S*_norm_ reaches maximum (*f*_max_) remain constant. Reducing the junction width has also been recognized as a common strategy in ionic bipolar diode design.^15,16^ In this circumstance,  increases with reduced *W*_m_, and Δσ_m_ should also increase because of ICP within the more confined space. This observation can be analogously applied to other system parameters, e.g., space charge density in polyelectrolyte gels and salt concentration in solutions (Figure 3e, scenarios iv and v). Similarly, a larger *N* or smaller *c*_0_ improves the ion selectivity of the P and N gels, thereby resulting in improved hysteresis with increasing Δσ_m_.

Incorporating the M gel in the middle of a bipolar diode is crucial for the memristor functionality. Figure S4 shows *I*–*V* hysteresis^42^ at 10 mHz in bipolar diodes without a M gel structure due to the development and disappearance of ICP at the membrane–microchannel interfaces.^43,44^ In the steady state, ions accumulated within the P and N gels at 1 V (Figure S4b), leading to a diminishing electrical potential drop across the polyelectrolyte gels (Figure S4c, compared to Figure S5g). The polyelectrolyte gels in memristors have larger electrical resistance compared to that associated with the P and N membrane section of the diode and the microchannels, hence governing the overall system resistance.

The geometrical effects of the three-layer P-M-N gel structure were thoroughly explored by focusing on the effect of the M gel length (*L*_m_) (Figure 3e, scenario ii) and P/N gel length (*L*_p_) (Figure 3e, scenario iii). The hysteresis area *S*_m_ did not monotonously decrease with *L*_m_ (scenario ii). Δ*R*_m_/*R*_total_ was reduced when *L*_m_ was decreased from 0.5 to 0.2 mm, which potentially diminishes the ion transport control by the hydrogel part of the fluidic system instead of the interfacing parasitic microchannel sections, especially when forward biased. As a result of the resistance reduction, the electrical potential drop was reduced over polyelectrolyte gels (Figure S5a) when compared with the potential drop for *L* = 0.5 mm (Figure S5g). At *L*_m_ = 2 mm, Δσ_m_ was significantly reduced compared to the original length *L*_m_ = 0.5 mm (Figure 3b), which can be confirmed by *c*(1 V)/*c*(−1 V) within the M gel at the steady state (Figure S5d). Under such conditions, modulation of ion enrichment and depletion was less efficient within the longer microchannel, also resulting in a reduced Δ*R*_m_/*R*_total_ and *I*–*V* hysteresis. Larger *L*_p_ consistently enhanced hysteresis performance (scenario iii) because the increase in the ion-selective membrane length enhances the ionic concentration polarization effect, leading to higher *c*(1 V)/*c*(−1 V) (Figure S5f compared to Figure 3b) and Δσ_m_ at the steady state within the M gel.

Ion transport within the P and N gels also contributes to the time-dependent responses of the system. It is hypothesized that, akin to unipolar memristors, the device length is a pivotal parameter that influences the memory time τ_m_. Results indicate that *f*_max_ monotonously decreases with the increase of *L*_m_ and *L*_p_ as expected due to the increased diffusion time. However, the changes of τ_m_ were notably less pronounced than predicted by diffusion within a homogeneous material (τ_m_ ∝ *L*^2^/*D*), as evidenced by scenarios ii and iii and also between the small and large memristors (Figure 3c,d). To assess the influence of microchannel impedance on the memristive effect, *L*_μ_ was reduced from 1 to 0.1 mm. In the original model (*L* = 1 mm), *R*_μ_ was close to *R*_total_/2 at forward bias (Figure S5g), while it was reduced to 1/10 of its original value in scenario vi. The results suggest that long microchannels reduce the hysteresis and that reducing *L*_μ_ emerges as an efficient strategy to improve memristor performance, especially at high scan frequencies.

### Ionic Memory and Neuromorphic Signal Processing

**Figure 4:**
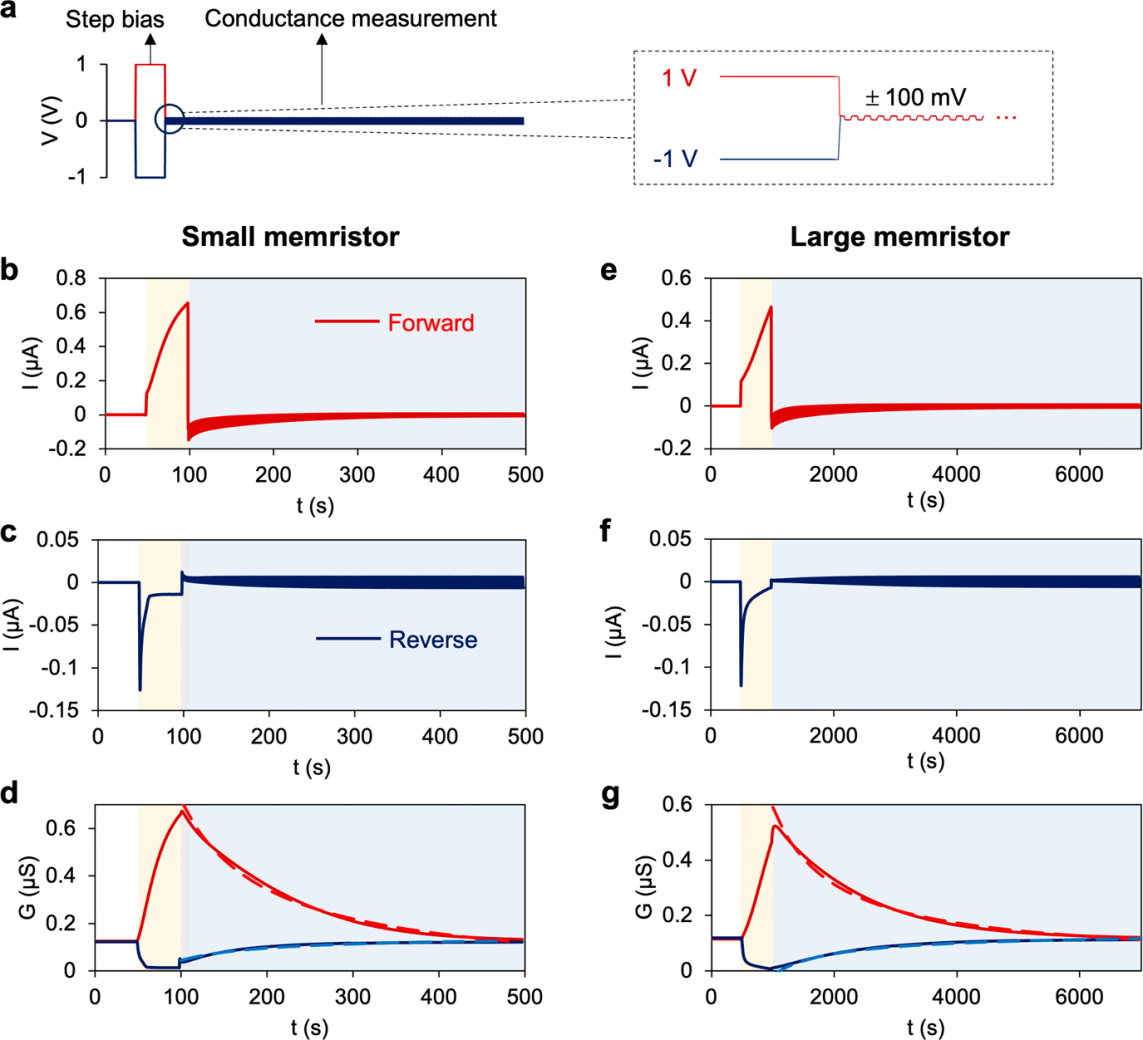
Experimental memory decay time tests. (a) Setup of the input voltage. After the rest period, a ±1 V step voltage was applied, and then, the AC voltage with an amplitude of 100 mV and frequencies of 1 and 0.1 Hz were employed to probe conductance of the small and large memristors, respectively. (b) Current–time response when the step voltage was 1 V in a small bipolar memristor. (c) Ion current response when the step voltage was −1 V. (d) Calculated differential conductance over time. (e–g) Ion current response and differential conductance in a large bipolar memristor. The dashed lines in parts (d) and (g) represent a semiempirical fitting of the conductance time response.

In this section, the potentiation and depression dynamics of the presented bipolar iontronic memristors was determined based on their response to a step voltage (Figure 4a). At the beginning, the system was in an equilibrium state with no bias voltage applied. Subsequently, a step voltage of ±1 V was applied, inducing a noticeable conductance change; the conductance was calculated as *G* = *I*/*V*. The conductance from 0 to 50 s was determined based on the value calculated at 50 s. Then, an AC read signal with a sufficiently low amplitude of 200 mV was utilized on the one hand to extrapolate the differential conductance by *G* = Δ*I*/Δ*V* but on the other hand was small enough to avoid significant ion flux across the polyelectrolyte, which would affect the memory of conductance. Careful selection of bias and read durations is critical to minimizing the imaginary component of electrical impedance. The AC electrochemical impedance spectroscopy (EIS) measurement indicated that the frequencies with minimal imaginary impedance ranged from 0.1 to 10 Hz, as shown in Figure S6. As a result, 1 and 0.1 Hz were chosen for the small and large memristors, respectively, in the following memory time measurements.

Figure 4b–g illustrates the temporal current response of the presented bipolar iontronic memristors to the designed voltage stimuli. Upon forward biasing, an ohmic current jump was initiated at ∼0.1 μA for both memristors. Then, this current significantly increased over the duration of the step bias due to continuous ion enrichment within polyelectrolyte gels. Comparing the relaxation of ion current at opposite polarities, the concentration change in the M gel was larger at 1 V than at −1 V (Figure 3b), indicating that more ions were crossing over the polyelectrolytes. In addition, the electric field across the polyelectrolytes was significantly larger at −1 V, attributed to the low electrical conductivity due to interfacial ion depletion. Consequently, the time for ion current to reach equilibrium at a step voltage of 1 V was longer than at −1 V, which has also been observed in the stepwise chronoamperometry measurement in Figure 2b. During the differential conductance measurement stage (i.e., >100 s), given that the mean AC voltage is 0 and the amplitude is small enough to avoid a hysteretic response, ion conductance decreases as ions that have been stored in a confined space within the polyelectrolyte gels diffuse out. The current obtained after turning off the applied voltage was an outcome of the diffusive flux and the transitioning from the step bias to the measurement mode. The calculated conductance recovered to the initial state (0 V) as the diffusive current diminished (Figure 4d,g). Such correspondence was also observed when the voltage was reverse biased, although in this case, the current depolarization and conductance recovery were due to ions diffusing from the microchannels into polyelectrolyte gels. The observed potentiation and depression of the ionic memory were numerically reproduced. Figure S7a,b shows the current response to voltage steps. Figure S7c–f details the corresponding ion concentration responses over time when the systems are biased and relaxed. The prolonged depression time in the bipolar systems due to diffusive ion dynamics, where ions are confined by ion-selective membranes, is consistent with the experiment measurements. The relaxation of conductance in Figure 4d,g was approximated by *G* ∼ *t*^–1^ exp(− 1/*t*) (as indicated by the dashed fitting curves), akin to the behavior in a unipolar charged nanochannel, as predicted by Fick’s law.^45^ Although sharing the same fundamental relaxation dynamics, the three-layer bipolar structure can sustain robust hysteresis due to a better control of ion transport, and the geometry can be easily scaled during fabrication.

**Figure 5:**
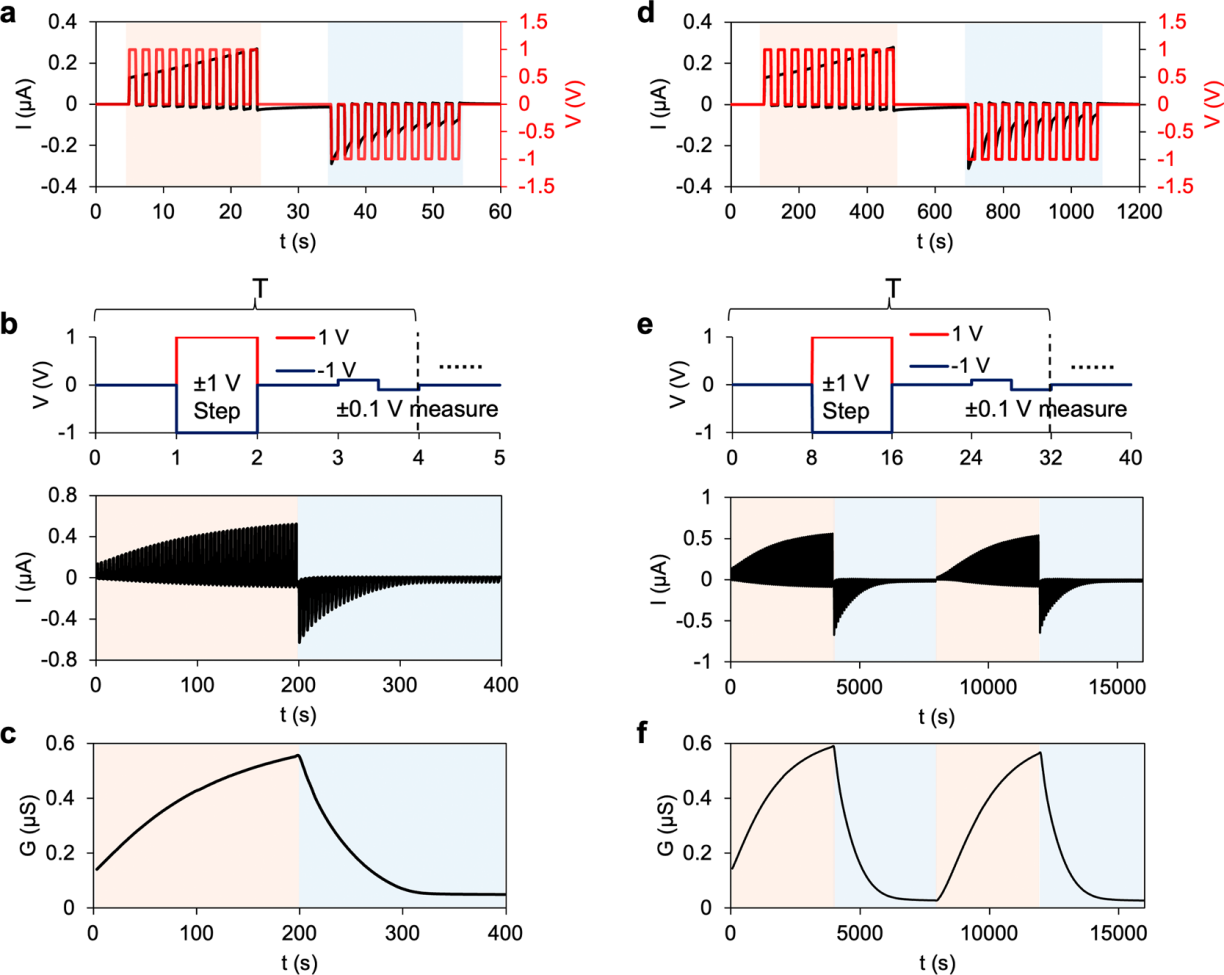
Pulse signal processing in iontronic bipolar memristors. (a) Evolution of the ion current (black) under voltage pulses (red) of constant polarity in a small bipolar memristor. (b) The upper panel shows a period (*T* = 4 s) of input voltage. The lower panel shows the ion current evolution under 50 set (1 V) and 50 reset (−1 V) pulses. (c) Differential conductance calculated from (b). (d,e) Same signal processing in a large bipolar memristor. (e,f) Containing one more period of conductance evolution in (b) and (c), where each period contains 125 set and 125 reset pulses.

The ability of the presented memristors to perform neuromorphic signal processing was explored by using pulse-based voltage inputs. In biology, the synaptic weights (i.e., strengths of connection) can be reversibly increased or weakened during a learning process by stimulation of a pulse voltage signal from a presynaptic neuron to a postsynaptic neuron, with memory time ranging from milliseconds to seconds and minutes to more than hours.^2,31^ The change of synaptic weights in the present memristors, which correlates with ion conductance in the polyelectrolyte gels, can also be tuned by voltage pulses. Figure 5a,d shows that the conductance of memristors can be continuously increased or decreased by a train of voltage pulses with the same polarity,^31^ representing neuromorphic potentiation and depression, respectively. Figure 5b, c, e, and f shows the prolonged conductance change over time when receiving a large number of voltage pulses as the input signal. The conductance was modulated by a ±1 V pulse and then measured as differential conductance by 0.1 and −0.1 V pulses in a period *T* and was repeated 50 and 125 times in the small and large memristors for each polarization cycle.

## Conclusions

In conclusion, this study demonstrated the realization of iontronic fluidic bipolar memristors with scalable memory times. The memristors were fabricated by using a low-cost and facile protocol that enables rapid prototyping of devices with diverse geometries. The normalized hysteresis area and memory time were harnessed to evaluate the performance of the memristors and provided insights into their fundamental ion transport mechanism, ionic memory capabilities, and neuromorphic signal processing. The memristive capacities were highly robust, significant, and reproducible and offer potential solutions to current challenges in memristor technology.^8^ The adjustable geometry enabled a wide range of memory times, from short-term memory to long-term memory regimes, and can be further reduced based on current fabrication methods. The non-negligible microchannel resistance, while presenting a challenge, can be mitigated by employing short and deep microchannel designs. The presented memristors are suitable for pulse signal processing because of their long memory time, multiple conductance states, and large hysteresis, which enable them to act as effective components for in-memory computing applications.^3,42^ Our next objective is to extend the planar memristors into small-scale integrated functional iontronic circuits that can implement intelligent algorithms with ions. Additionally, it is promising to build memristors using hydrogels with ion specificity, such as crown-ether and aptamers.^46,47^ Such developments can provide possibilities to realize systems that can differentiate specific ion types, much like biological ion channels. This approach can mark a significant step toward leveraging the advantages of iontronics, particularly the use of multiple ion species, for ionic computation. Future research should focus on integrating more biomimetic neuromorphic functionalities into artificial micro-/nanofluidic systems, further bridging the gap between biology and iontronic technology.

## Methods

### Fabrication

A pair of soda lime glass slides (1 mm thickness, Marienfeld) were used as the substrate for the microchannels (Figure S2). A hand driller was used to drill Φ 1 mm holes into one of the slides to obtain a microfluidic inlet and outlet. The glass slides were immersed in piranha solution (98% H_2_SO_4_:30% H_2_O_2_ = 3:1, J.T.Baker) for 10 min at 120 °C followed by deionized water washing and hot-plate baking at 120 °C for 10 min. Then, methanol solutions containing 0.5% TMSMA (3-(trimethoxysilyl)propyl methacrylate, Sigma) and 0.5% glacial acetic acid were used to treat the glass slides for 90 min. The slides were then coated to ensure the hydrogel adhesion to the substrate. A 3M optical clear adhesive (OCA) (3M optically clear adhesive 8146-1-ND, 25 μm) was cut with a femtosecond laser cutter (ELAS Master Femto) following a computer-designed shape. A low-power green laser source was chosen to scan the cutting trajectory hundreds of times to ensure that the cutting edge of the OCA was not burnt but thoroughly cut. After cleaning the patterned OCA with isopropyl alcohol, it was carefully transferred to a clean glass and sandwiched by another glass to form closed microfluidic channels. Custom-made photomasks were aligned with microfluidic channel patterns. A mixture of 50% PEGDMA monomers (Mn 550, Sigma) with a 2% photoinitiator (2-hydroxy-4′-(2-hydroxyethoxy)-2-methylpropiophenone, Sigma) and 50% 10 mM KCl was injected into the microchannels. The pPEGDMA gel was first patterned by exposing it to UV light (21 mW/cm^–2^) for 90 s. The microchannels were then washed 3 times, and stored in solution for several hours. Then, anionic and cationic gels were patterned sequentially. Diallyldimethylammonium chloride (DADMAC, Sigma, 4.2 M) and 5 M 2-acrylamido-2-methyl-1-propanesulfonic acid (AMPSA, Sigma) are monomers of anionic and cationic polyelectrolyte gels. Each monomer was mixed with a 2% (w/w) photoinitiator (2-hydroxy-4′-(2-hydroxyethoxy)-2-methylpropiophenone) and a 2% (w/w) cross-linker (*N*,*N*′-methylenebis(acrylamide), Sigma). The UV exposure power was 10 mW/cm^–2^, and the exposure times were 10 s for DADMAC and 20 s for AMPSA. After hydrogel formation, the chips were stored in 10 mM KCl solutions for 2 days for hydrogel swelling.

### Measurements

KCl 10 mM solutions (1.61 mS/cm^–1^) were used as bulk solutions in all of the experiments. A source meter (Keithley 2636A) and Ag/AgCl electrodes (A-M Systems, 0.015”) were used for electrical characterization of bipolar memristors. All *I*–*V* scan and chronoamperometric measurements were realized by connecting the source meter and the microfluidic chip through electrodes inserted in the reservoirs. In all *I*–*V* scans, the voltage was set to −1 V for a sufficient time until saturation was reached, and then, the scan from −1 to 1 V was initiated. The EIS results were obtained using a compact potentiastat (Bioanalytics Palmsens 4). The optical images were obtained through a spinning disc confocal system (Yokogawa CSUX1) and an inverted microscope (Eclipse Ti–U, Nikon) equipped with an electron-multiplying charge-coupled device (EMCCD) and a camera (Andor iXon3). The anionic dye solution was composed of 1 μM DyLight 488 (Thermo Scientific, Inc.) in 10 mM KCl solution.

### Numerical Simulations

All of the numerical simulations were conducted by COMSOL Multiphysics 5.3a. A simplified one-dimensional model was constructed to reproduce the ion transport phenomena observed in experiments (Table S1). The ion-selective membranes were modeled with fixed space charges while ignoring the possible difference in the diffusion coefficient compared to that in bulk due to molecular mechanisms such as sieving and adsorption. The whole space was described by Poisson–Nernst–Planck equations:
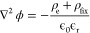
1
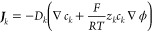
2
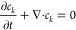
3where ϕ is the electrical potential, ρ_e_ is the mobile space charge density, ρ_fix_ is the fixed space charge density, ϵ_0_ is the vacuum permittivity, ϵ_r_ is the relative dielectric constant, *t* is time, *k* is the ion type, ***J***_*k*_ is the ion flux density, *D*_*k*_ is the diffusion coefficient, *c*_*k*_ is the ion concentration, and *z*_*k*_ is the ion valency. To consider the microchannel width in the 1D model, the diffusion coefficients were replaced by effective diffusion coefficients due to the continuity of ion flux:
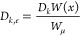
4where *W*(*x*) is a function of the microchannel coordinate *x*. As shown in Figure 3a, the constant electrical potential and concentration were set at the boundary of the microchannels as boundary conditions. The fixed space charge density was set to a low value (20 mM) to ensure convergence of the numerical calculation. It underestimates the memristive hysteresis due to less effective rectification of ion concentration. The ion current was calculated at certain locations in the microchannels as *I* = *H*_μ_*W*_μ_Σ_*k*_*Fz*_*k*_***J***_*k*_, where *H*_μ_ is the microchannel height.

## Supporting Information Available

Additional Figures S1–S7 and Table S1 that enhance the understanding of microchannel geometries, fabrication process, additional experimental *I*–*V* response curves, numerical modeling of bipolar diodes, numerical modeling of the effect of the polyelectrolyte gel length, Bode phase plot of the EIS experimental measurements, and numerical simulations of the temporal response to voltage steps (PDF)
