# Supplementary material for: Geometrically Scalable Iontronic Memristors: Employing Bipolar Polyelectrolyte Gels for Neuromorphic Systems
Source: ACS Nano. 2024 May 28;18(23):15025–34. doi: 10.1021/acsnano.4c01730 (PMC11171754; doi:10.1021/acsnano.4c01730)
Supplement: Supplementary file 1 — nn4c01730_si_001.pdf [file nn4c01730_si_001.pdf]

Supporting Information:

Geometrically Scalable Iontronic Memristors: Employing Bipolar Polyelectrolyte Gels for Neuromorphic Systems

Zhenyu Zhang[1,2], Barak Sabbagh[1,3], Yunfei Chen[2], Gilad Yossifon[1,4*]

[1] *School of Mechanical Engineering, Tel-Aviv University, Tel Aviv 6997801, Israel*

[2] *Jiangsu Key Laboratory for Design and Manufacture of Micro-Nano Biomedical Instruments, School of Mechanical Engineering, Southeast University, Nanjing 211189, China*

[3] *Faculty of Mechanical Engineering, Technion−Israel Institute of Technology, Haifa 3200003, Israel*

[4] *Department of Biomedical Engineering, Tel-Aviv University, Tel Aviv 6997801, Israel*

[*] *Corresponding author. Email: gyossifon@tauex.tau.ac.il*

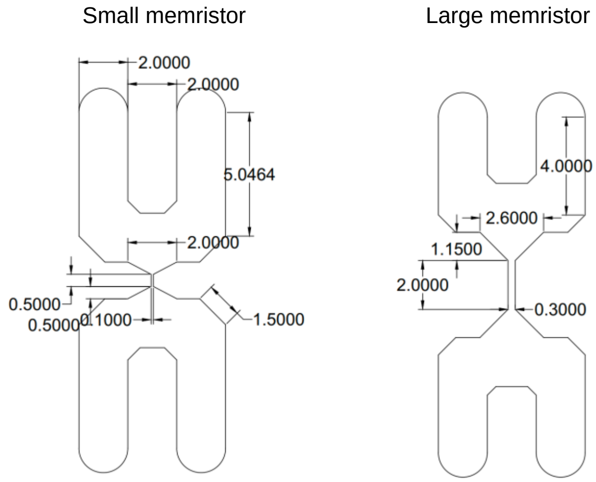

Figure S1. Geometrics of microchannels designed for the small and large memristors.

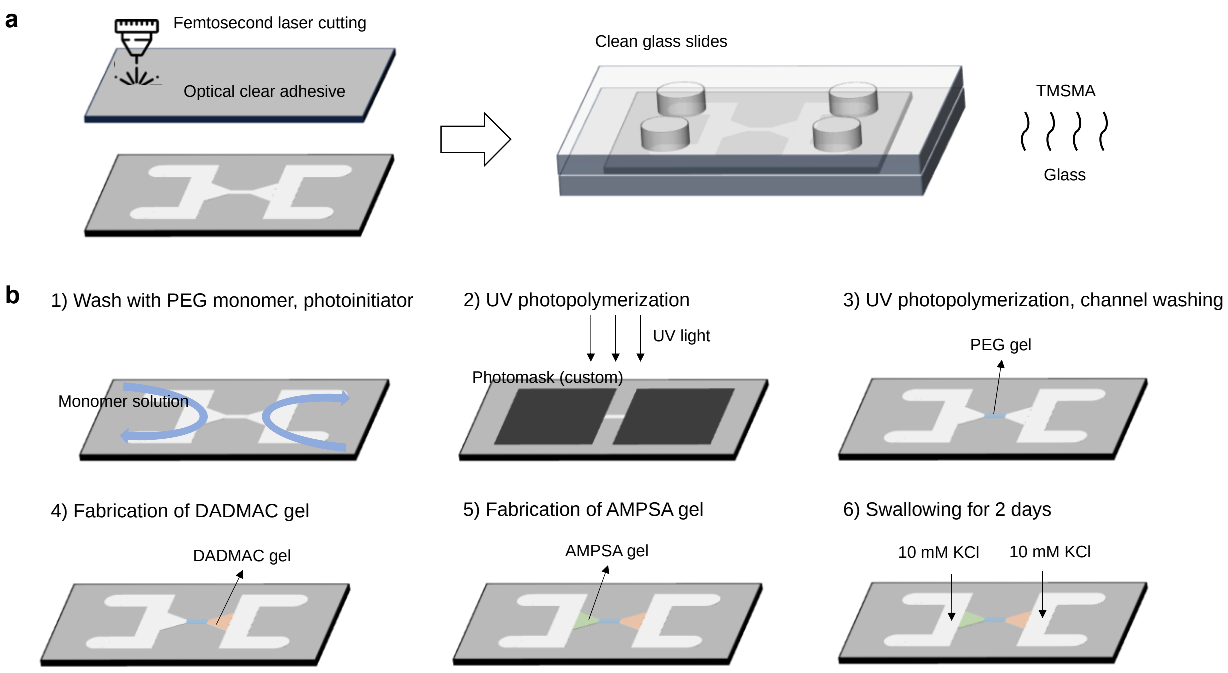

Figure S2. Fabrication process of iontronic bipolar memristors. (a) Fast prototyping of microfluidic chip. First, an optically clear double-sided adhesive (25 μm) was cut with a femtosecond laser and sandwiched between two glass slides. Then, the glass microchannels were chemically modified by TMSMA. (b) Fabrication of three layers of hydrogels by UV photopolymerization.

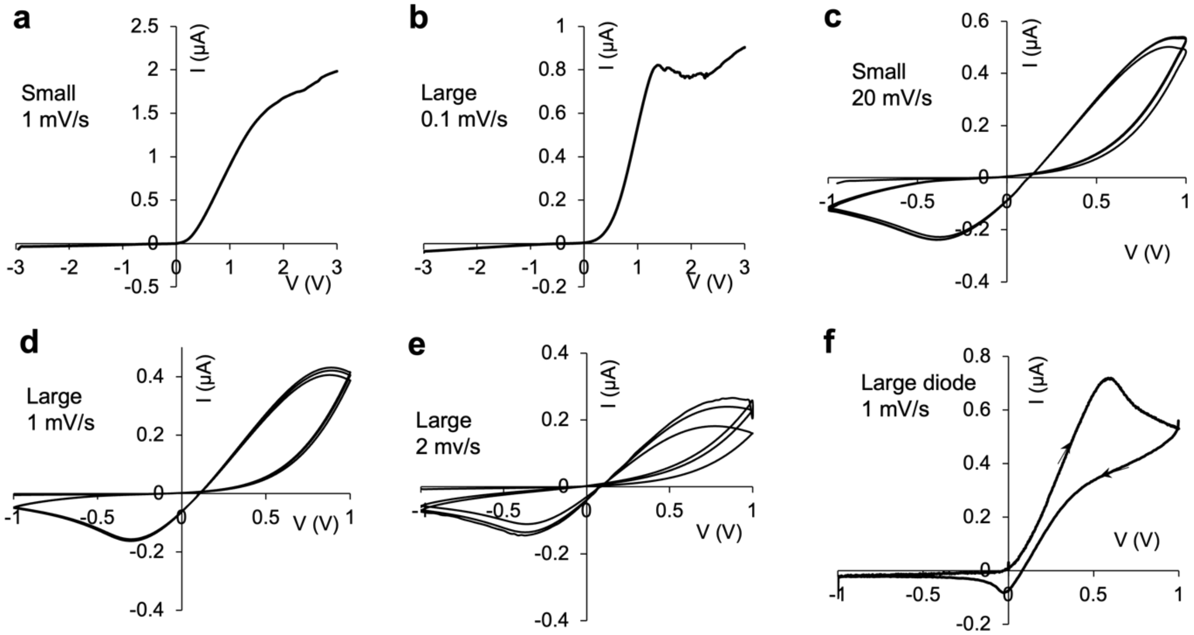

Figure S3. Experimental I-V curves. I-V curve of (a) the small memristor at a scan rate of 1 mV/s and (b) the large memristor at a scan rate of 0.1 mV/s. The scan rate was low enough to avoid hysteresis, thus the results in (a) and (b) represent steady-state responses. (c), (d) and (e) show the I-V curves including the data from when the scans began and then reached quasi-stable hysteresis loops. (f) The I-V curve of a bipolar diode at a scan rate of 1 mV/s. The ionic diode features the same geometry as the large memristor except for the middle neutral layer (M gel).

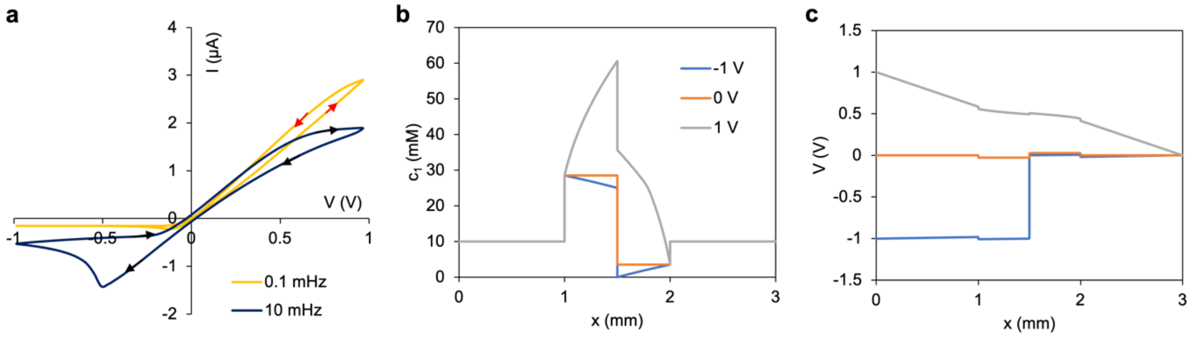

Figure S4. Numerical modeling of bipolar diodes. (a) I-V curves at 0.1 and 10 mV/s. The arrows indicate the directions of the voltage scan. (b) The steady-state cation concentration distribution profiles at different applied voltages. (c) The electrical potential distributions at different applied voltages.

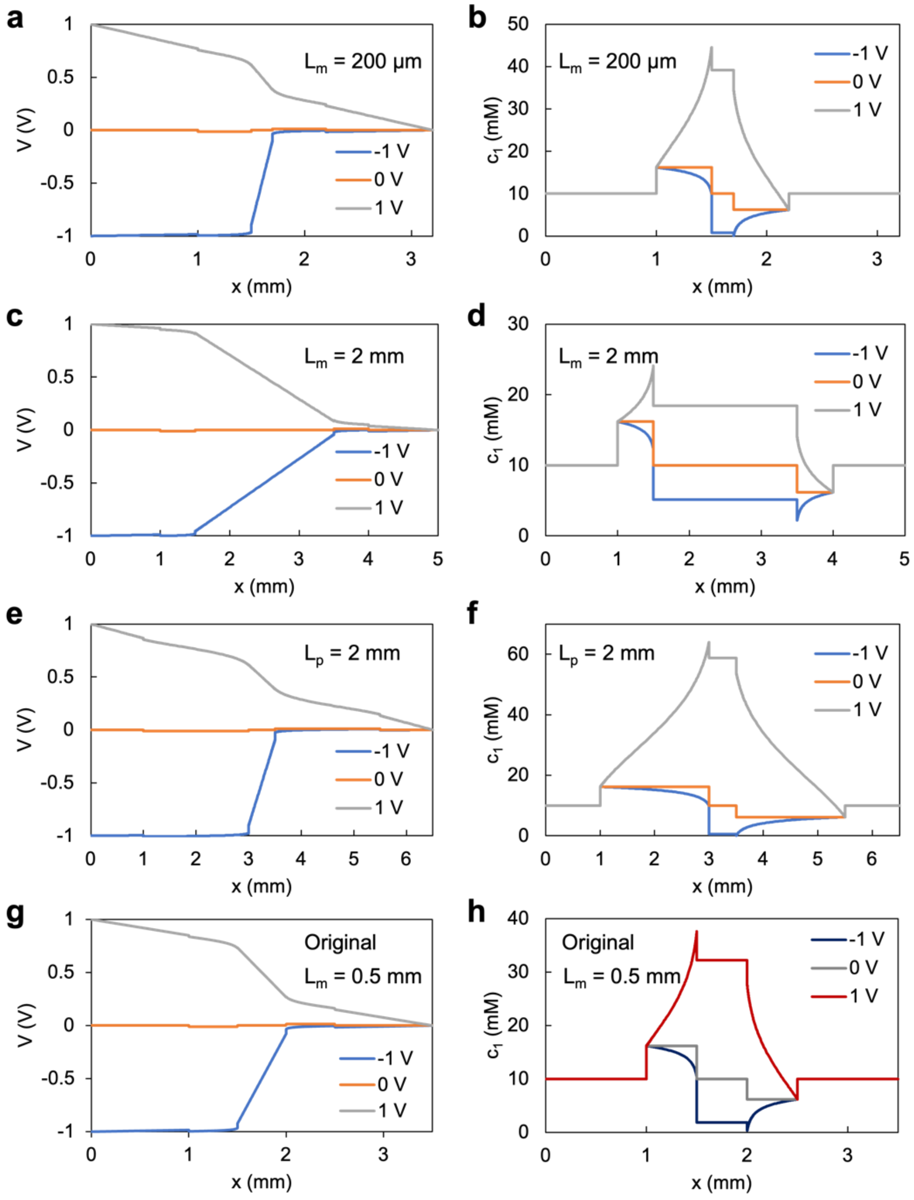

Figure S5. Numerical modeling of the steady-state responses as a function of polyelectrolyte gel length. (a), (c), (e), (g) show the electrical potential distributions, and (b), (d), (f) show ion concentrations with bias voltage of -1 V, 0 V and 1 V.

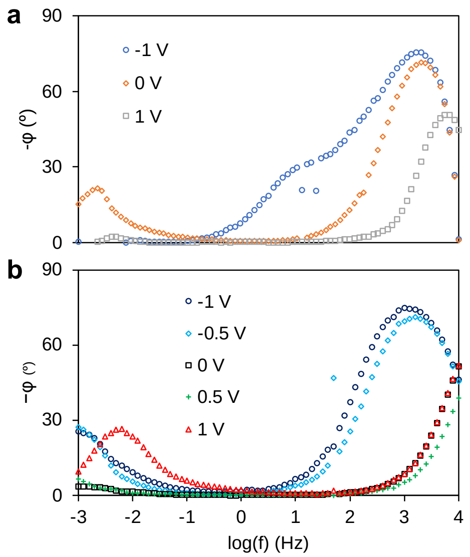

Figure S6. Bode phase plot of electrical impedance spectroscopy measurement in (a) the small and (b) the large memristor, with DC voltage ranging from -1 V to 1 V. The DC bias voltages were from -1 V to 1 V, and AC voltage was 200 mV. The AC voltage frequencies ranged between 1 mHz and 10 kHz.

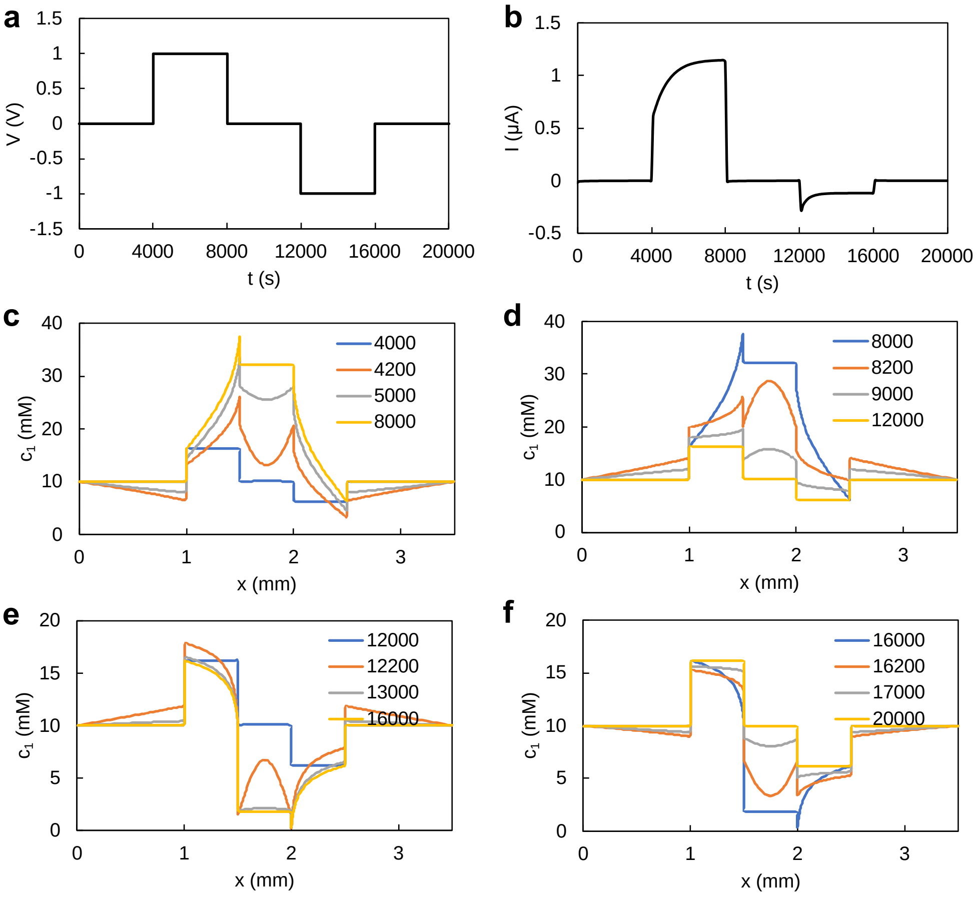

Figure S7. Numerical simulations of ion current and concentration responses to voltage steps. (a) The voltage input. (b) The calculated current over time. (c)-(f) Ion concentrations at different times in (a).

Table S1. Variables in numerical simulations

Variable	Value	Description
c_0	10 [mM]	Concentration in bulk solutions
w_μ	2 [mm]	Microchannel width
w_m	100 [μm]	Middle channel width
L_μ	1 [mm]	Microchannel length
L_p	0.5 [mm]	Polyelectrolyte gel length
L_m	0.5 [mm]	Middle channel length
N	20 [mM]	Fixed space charge molar density
V_{app}	1 [V]	Voltage amplitude
